# Improving the Quality of Laser Drilling by Assisted Process Methods of Static Solution and Mist Blowing

**DOI:** 10.3390/mi15040515

**Published:** 2024-04-12

**Authors:** Yuan Tao, Zhiwei Wang, Shanshan Hu, Yufei Feng, Fan Yang, Guangliang Li

**Affiliations:** 1 College of Mechanical Engineering, Guangxi University, Nanning 530004, China; chersonese1@163.com (Y.T.); 2111391024@st.gxu.edu.cn (Y.F.); youngfine@mail.dlut.edu.cn (F.Y.);; 2 College of Mechanical and Electronic Engineering, Shandong University of Science and Technology, Qingdao 266590, China; zwwang@live.com; 3 School of Mechanical Engineering, Dalian University of Technology, Dalian 116024, China

**Keywords:** laser drilling, alumina ceramic, blowing method, solution composition, drilling quality

## Abstract

The use of static solution-assisted laser drilling can effectively improve hole roundness, decrease taper angle, and reduce recast layer thickness and hole wall slag adhesion. However, the enormous energy of the laser will evaporate the solution to form a suspension droplet and reduce the quality and efficiency of laser drilling. To deal with this defect, the mist-blowing method was used to reduce the influence of droplets on the taper angle and recast layer. In this work, the effect of wind speed on drilling quality was examined, and laser drilling in air, water, and NaCl solution was carried out to analyse the effect of solution composition on hole wall morphology. The results showed that a speed fan with a proper wind speed that disperses the droplets formed in the processing area can significantly reduce the refraction and scattering of the laser, and the taper angle and roundness of the drilling hole were also reduced by 15.6% and improved by 2.4%, respectively, under the wind speed of 2 m/s. The hole wall morphology showed a thicker recast layer and cracking in air, while it was thinner in water and there was little or no layer in the NaCl solution in the same current. When drilling in NaCl, the taper angle and roundness of the drilling hole were reduced by 4.13% and improved by 2.11%, respectively, compared to water. Due to the mechanical effect of the laser in the NaCl solution, the impact force on the material was much greater than that in water. The solution cavitation effect, generated by the absorption of laser energy, caused an explosive impact on the molten material adhered to the surface of the hole wall. Above all, drilling in the NaCl solution with a current of 200 A and a wind speed of 2 m/s was the optimal condition for obtaining the best processing quality.

## 1. Introduction

Ceramic is widely used in modern industry fields such as aerospace, bulletproof vests, biomedicine, and electronic information [1,2,3,4,5] because of its excellent characteristics of high hardness, high temperature, and corrosion resistance. It is also a typical hard-processing material due to its high strength and brittleness. Laser drilling can effectively eliminate the contact stress of conventional ceramic drilling. However, in the process of laser drilling, other problems often appear, such as large taper angles, thick recast layers, hot cracks, and slag [6,7,8,9,10].

Changing the type of laser is one of the ways to solve the above problems. Min [11] used dual femtosecond laser scanning to micro-process porous alumina ceramic for a turbine blade. The results showed that the cutting quality was related to the cutting depth, width, and efficiency. By utilising the cold processing characteristics of the femtosecond laser, there was almost no obvious solidified material recast layer on the surface of the processed alumina ceramic. Surendra’s study [12,13] confirmed that an Nd: YAG laser could be used for drilling difficult-to-process alumina ceramic materials. They found that a low pulse frequency and high auxiliary gas pressure could slow down the formation of the recast layer and microcrack defects, as well as minimise the change in the microhardness of the ceramic under the processing conditions. Zhang [14] proposed the processing method of combining laser drilling with jet electrochemical machining to address defects such as molten spatter and recast layers in laser drilling. The research demonstrated that, compared to drilling in air, the new process could effectively reduce the generation and splashing of recast layers. Jia [15] conducted a numerical model analysis of composite pulse laser (CPL) drilling in alumina ceramics; the results showed that the drilling performance of a millisecond laser could be improved by increasing laser absorption or reducing melt sputtering.

Because the direction of gas injection is opposite to the direction of slag splitting during gas-assisted laser drilling, some slag still adheres to the processing area. The surface quality of the ceramics drilled in air is greatly affected by thermal stress. Therefore, scholars have proposed experimental studies for laser drilling assisted by water and other solution media. Reference [16] focused on the thermal damage to zirconia during laser drilling and reported an experiment using water solution-assisted ultrafast laser drilling. The results showed that laser-target coupling could enhance the interaction between the laser and the processed material in water, including limiting heat accumulation and removing the molten materials, which helped reduce the recast layer and thermal stress. Zahrani [17] analysed the morphology of single-point laser ablation holes in silicon nitride in air and water solutions by taking the focal position and underwater depth into account. The conclusions showed that the depth and diameter of ablation, hole taper angle, and roundness all changed with the aforementioned process parameters. Liang [18] summarised recent research into the laser drilling of alumina ceramic. The principle of millisecond laser drilling indicated that, due to the large amount of laser energy injected, the material exhibited significant thermal effects throughout the entire process from heating to melting, which would produce molten material and a recast layer. When drilling in a solution medium, the molten material was carried away by saturated vapour pressure or water vapour, and the thermal effect of drilling was reduced or even eliminated. Ref. [19] drilled alumina ceramic using a picosecond laser in water solution and proposed that the expansion of the hole’s size in a water solution, compared to air, was due to the scattering effect of water on the laser beam. As a high-power density laser, a picosecond laser could also cause cavitation in liquids; the collapse of bubbles had a strong impact on the hole wall, which helped to detach and discharge the recast layer. Ren [20] conducted femtosecond laser-drilling experiments on alumina ceramic, assisted by a water solution, and found that the residual ablative material on the bore surface reduced. In the meantime, there was almost no slag attachment near the hole outlet when drilling in water, which could achieve better quality compared to drilling in air. Moreover, the solution flow caused by bubbles and the increase in plasma recoil pressure in water could significantly improve the material removal rate. In a femtosecond fibre laser-drilling experiment on SiC ceramic sheets [21], the microfluidic effect of the water solution reduced the overall thermal stress by homogenising the transient temperature gradient. The local bubble cavitation effect of water near the hole wall could also effectively reduce the thermal stress and improve the surface quality and uniformity of the bore surface.

In addition to the use of water as a medium, other electrolyte solutions for assisted laser drilling have also had significant effects. Research has been conducted by [22], in the form of an electrochemical post-treatment experiment, using a NaNO_3_ and NaCl solution to investigate the characteristics of Inconel 718 laser drilling. It was found that neutral salt solution-assisted laser processing combined the thermal effects of a laser and the thermochemical effects of a salt solution at high temperatures. The thermal effects of a laser were used for rapid material removal, while the cooling and thermochemical effects of a salt solution were used for removing recast layers and heat affected zones. Due to the absorption and scattering effects of a salt solution on lasers, the transmission of a laser in a salt solution was attenuated. Compared with pure water, the attenuation coefficient of a laser in a NaCl solution was higher. The change in salt solution concentration had a significant impact on the removal of the recast layer, while the change in flow rate mainly influenced the reduction in the heat-affected zone [23].

Although the composite laser processing or solution-assisted laser-drilling methods mentioned in the above literature can effectively improve the hole quality, it was found that the water solution evaporated and condensed to form droplets on the laser lens by the laser beam. The accumulating droplets and mist refracted and scattered the laser beam and then expanded the spot area, which greatly affected the quality of drilling [24,25]. The introduction of a mist-blowing method may simultaneously cool down the solution and disperse suspended droplets to reduce laser refraction. Therefore, we proposed a different method of solution-assisted laser drilling by mist blowing in this work. By studying the process parameters of the mist-blowing method with different solution compositions, the optimal processing parameters to improve hole roundness and reduce the hole taper were pursued. At the same time, the morphological differences in laser drilling in air and different solutions were observed and compared to analyse the underlying causes and to give better processing parameters.

## 2. Materials and Methods

The experiment was performed using a HYM-750-type Nd: YAG solid-state laser machine produced by Beijing Hairun Chuangda Laser Technology Co., Ltd. (Beijing, China), with a laser wavelength of 1064 nm and a current output power of 12 kW. A beam with a spatiotemporal Gaussian distribution was formed by the optical system. Its maximum single pulse energy was 120 J, and the current could be continuously adjusted between 100 and 400 A with accuracy of 1 A. The laser frequency was 1–100 Hz, and the pulse width was 0.1–15 ms. A variable speed fan with a wind speed range of 0–4.5 m/s was used and placed 50 mm away from the laser outlet, and it was adjusted to be level with the laser outlet to ensure that water mist was blown parallel.

**Figure 1:**
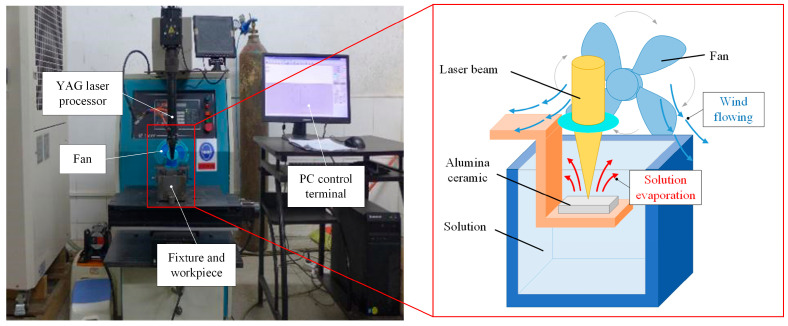
Experimental platform and schematic diagram of laser drilling by mist-blowing method.

The work piece was alumina ceramic with a purity of 96% and a size of 15 × 15 × 1 mm^3^. Its surface was polished before the experiment to prevent surface defects from affecting the experiment, and then cleaned with anhydrous ethanol. The experimental platform and schematic diagram are shown in Figure 1.

**Figure 2:**
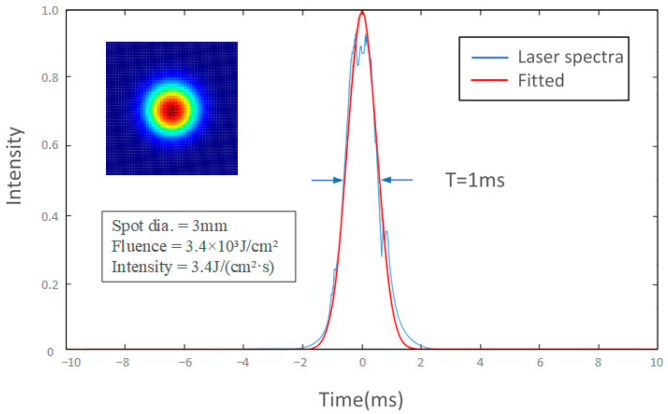
Laser pulse temporal profile.

A fixed-point drilling method was used to conduct laser-drilling experiments on alumina ceramic sheets. Through preliminary research, the most suitable laser parameters under the condition of a water solution could be determined as current I = 200 A; laser pulse width T = 1 ms; and laser frequency f = 40 Hz. The range of wind speed was from 0 to 4.5 m/s with intervals of 0.5 m/s. The experiment was repeated 4 times for each group. The morphology of the upper and lower surface of the ceramic sheets was observed and measured by 4XC-NS metallographic microscopy and Phenom pure+ scanning electron microscopy. The laser parameters and experimental parameters are shown in Table 1 and Table 2 and Figure 2.

## 3. Results and Discussion

### 3.1. Influence of Mist-Blowing Method on Hole Quality

#### 3.1.1. Taper

During the laser-drilling process, the taper θ of the hole was calculated by the diameters of the light-in hole (upper hole) $d_{1}$ and light-out hole (lower hole) $d_{2};$ thus, it was calculated as follows:

(1) $$\tan\frac{\theta }{2}=\frac{\left(d_{1}-d_{2}\right)∕2}{h}=\frac{d_{1}-d_{2}}{2h}$$

(2) $$\theta =2arctan(\frac{d_{1}-d_{2}}{2000})$$

where h is the thickness of the ceramic material.

**Figure 3:**
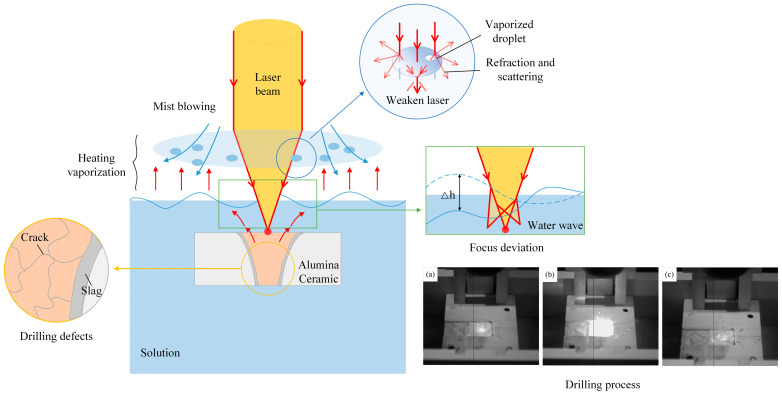
Mist-blowing laser-drilling process in water. (Drilling process of one laser pulse was recorded from image (**a**–**c**)).

As is shown in Figure 3, when drilling ceramic in solution, the injection of a laser causes a portion of the solution above the ceramic sheet to evaporate, which accumulates and becomes liquid droplets suspended in the air. The laser beam needs to pass through the droplets, increasing the scattering of the laser and reducing the quality of drilling.

**Figure 4:**
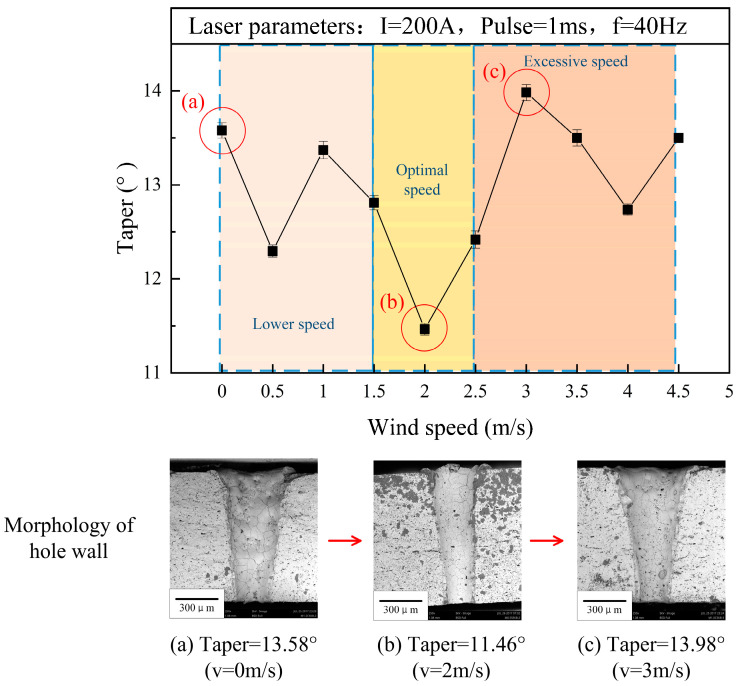
Influence of wind speed on hole taper angle (drilling in water).

Figure 4 shows the influence of wind speed on the hole taper angle. The curve of taper angle θ and its variation with wind speed v showed a non-monotonic trend. When there was no wind blowing (v=0), the hole taper was 13.58°. At a low wind speed of 0.0–1.5 m/s, the blowing effect of wind on the suspension droplets was not significant, so the taper of the hole hardly changed with the increase in wind speed. As the wind speed reached 2 m/s, the hole taper decreased to the minimum value of 11.46°. At this time, the liquid droplets were basically dispersed and the energy of the laser beam was less weakened, leading to the stronger penetration ability of the laser, which helped in the vertical drilling of the hole. Until the wind speed increased to 3 m/s, the taper reached its maximum value of 13.98°, which was even larger than that when drilling without wind blowing. The hole taper remained high at speeds in excess of 3.0–4.5 m/s; this was because the wind dispersed the suspension droplets and the laser energy directly irradiated on the solution and ceramic surface. However, the energy-absorbing solution accelerated the generation of water vapour and the laser scattered and refracted again, which was reflected in the increase in the hole taper. At the same time, the solution fluctuated with stronger wind speeds, also increasing laser scattering. Therefore, only specific wind speeds had a positive effect on the taper.

#### 3.1.2. Roundness

The ratio of the minimum and maximum diameters (*D*_2_ and *D*_1_) on the cross-section of the hole defines the ‘hole roundness’, as Equation (3) describes as follows:

(3) $$R=\frac{D_{2}}{D_{1}}\;(\%)$$

**Figure 5:**
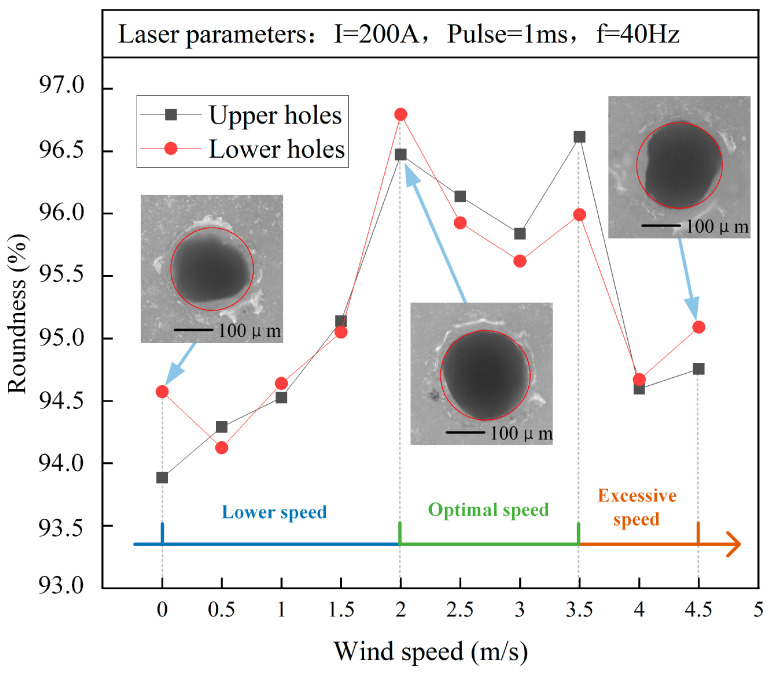
Influence of wind speed on the roundness of upper and lower holes (drilling in water).

Figure 5 shows the influence of wind speed on the roundness of the upper and lower holes. The relationship between roundness R and wind speed v showed an overall trend of first increasing and then decreasing, and the roundness of the upper and lower holes changed consistently with wind speed. Obviously, the assistance of the mist-blowing method can significantly improve laser-drilled hole roundness. The wind speed range for obtaining optimal roundness was 2.0–3.5 m/s, which partially overlapped with the range of the optimal taper of 1.5–2.5 m/s. When the wind speed was 2.0 m/s, the roundness of the lower and upper hole reached its highest and second highest value, respectively. When v≤2 m/s, the wind speed was not fast enough to fully disperse the suspended droplets and evaporated mist, which would cause the laser beam to scatter more slowly. When v≥3.5 m/s, as the wind speed increased, the suspension droplets were blown away and the solution was fluctuated by the wind, accelerating the solution’s heat dissipation. In this case, the increase in laser scattering would prevent the laser energy from being concentrated at the drill point, resulting in a decrease in the roundness value of the hole.

#### 3.1.3. Morphology Quality of Holes

**Figure 6:**
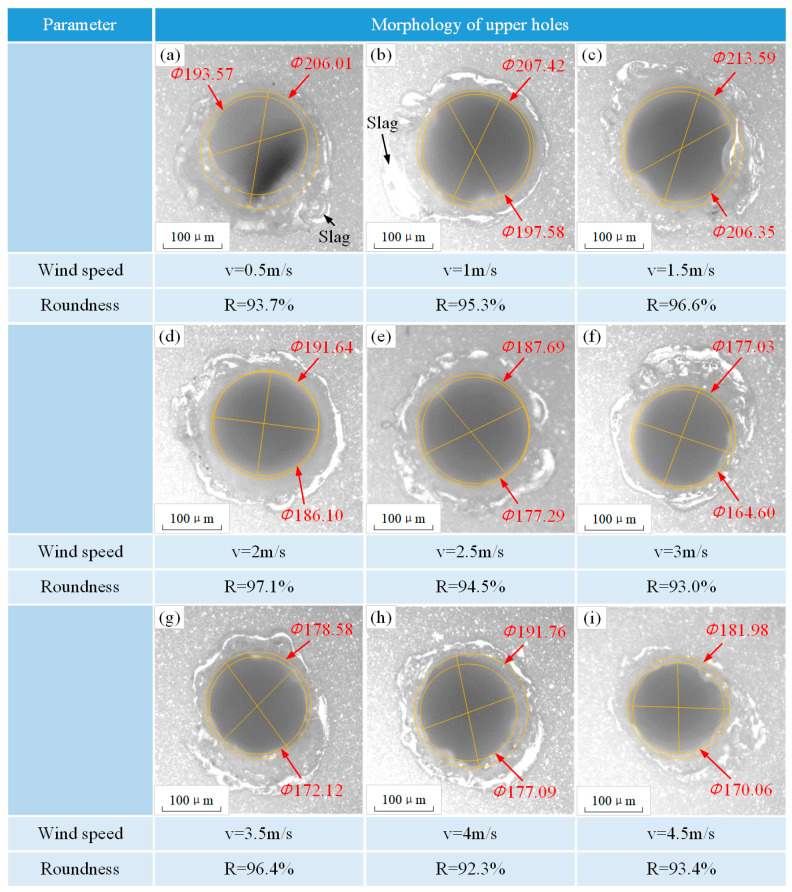
Metallographic morphology of alumina ceramics at different wind speeds (drilling in water). The morphologies (**a**–**i**) are under the conditions of small wind speed to large wind speed.

Figure 6 shows the metallographic morphology of alumina ceramics at different wind speeds. In Figure 6d, the accumulated slag at the surface was significantly lower and less uniform at a wind speed of 2 m/s, and its roundness reached the maximum value. The proper use of wind speed could dramatically improve the drilling quality. As the wind speed increased, the slag accumulated on the upper surface of holes and began to increase, as shown in Figure 6f,h. Higher wind speeds increased the fluctuation amplitude of the solution, which had a certain impact on the hole quality. The molten material began to detach from the inner wall under the influence of the faster flow of liquid and reattached to other parts of the inner wall, forming new slag. The thicker slag caused a decrease in hole roundness and reduced the quality of drilling.

### 3.2. Influence of Drilling Environments on Hole Quality

From the above study, the optimal wind speed was found to be v=2 m/s. An auxiliary experiment was carried out to laser drill alumina ceramic at a wind speed of v=2 m/s in water and NaCl solutions to ensure the influence of drilling environments on hole quality.

#### 3.2.1. Taper

**Figure 7:**
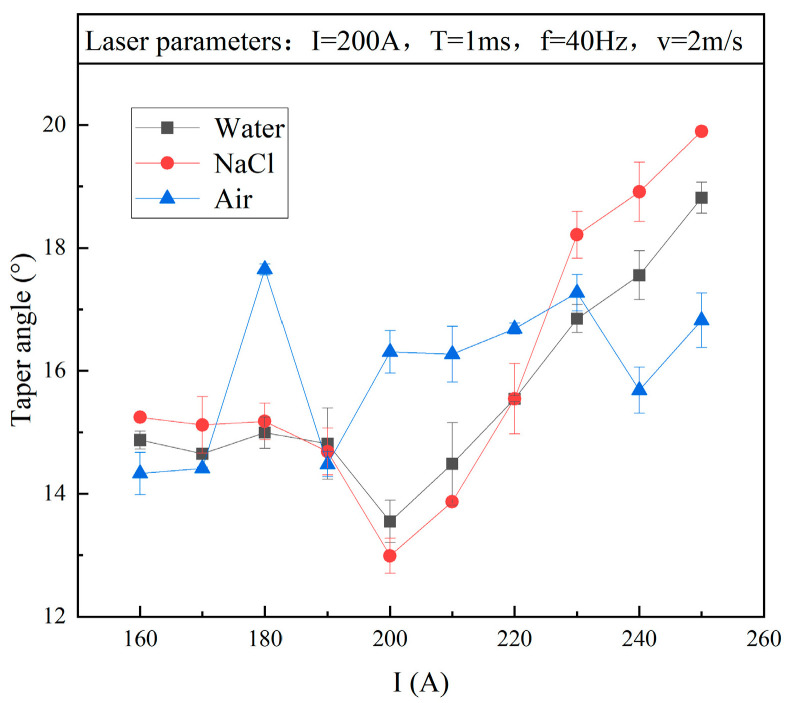
Influence of different environments on taper: (1) water; (2) NaCl solution; (3) air.

Figure 7 shows the influence of electric current on taper angle. When the current I = 200 A, the taper angle in both the water and NaCl solution reached the minimum value, which was the optimal drilling quality under this current parameter. As the current continued to increase, the taper of the hole rapidly increased. The reason for this was that the excessive current made the laser energy too high, which caused a large amount of water vapour in the solution. Then, the laser was refracted and scattered, expanding the diameter of the upper holes. Therefore, the hole taper increased and the drilling quality decreased. At the same time, when the current was in the range 160–190 A, the hole taper did not change significantly and the overall trend was relatively gentle, which indicated that the current was too small to change the taper of the hole, whether in water or NaCl solution.

Comparing the hole taper in the water and NaCl solution when I ≤ 180 A or I ≥ 220 A, the drilling in the water solution achieved a smaller taper. However, when the current was in the ideal range of 190–210 A, drilling in NaCl solution produced a smaller hole taper. The reason for this was that, within this current value range, the NaCl could prevent the adhesion of slag on the inner wall and surface of the workpiece and obtain an ideal hole taper due to the reduced refraction and continuous cooling effect.

#### 3.2.2. Roundness

**Figure 8:**
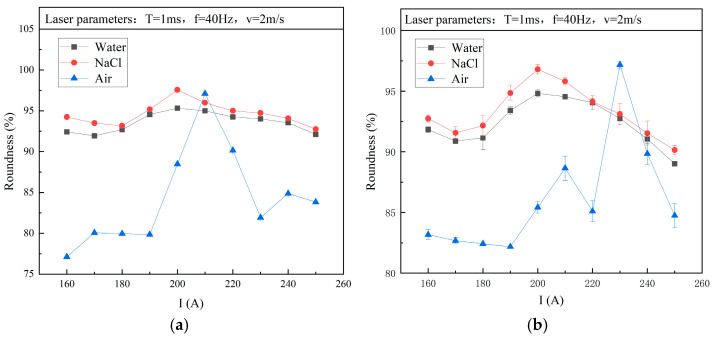
Influence of current on roundness (drilling in water, NaCl solution, and air). (**a**) Upper hole; (**b**) lower hole.

The influence of current on the roundness of holes in air, water, and NaCl solutions is shown in Figure 8, and I = 200 A was found to be the optimal current value for obtaining the best processing quality. After exceeding 200 A, as the current continued to increase, the roundness of the upper and lower holes rapidly decreased. The reason for this change was that the excessive current caused higher energy, which made it difficult to control the melting of the ceramic material during the drilling process. This resulted in irregular hole shapes and reduced hole roundness. When the current was in the range of 160–200 A, the roundness of the upper and lower holes first decreased and then increased, which was due to a lack of laser energy available to melt and drill through the hole with high quality.

Comparing the roundness curves of the upper and lower holes in the water and NaCl solution, the trends of both solutions were basically the same. However, from the roundness values corresponding to the same current, drilling in the NaCl solution could achieve better roundness. The boiling point of the NaCl solution was higher than that of water, so it was not easy to evaporate and produce suspension droplets. In addition, the blowing mist effect jointly reduced the uneven refraction of the laser towards the surrounding side, thereby improving the roundness of the hole.

#### 3.2.3. Morphology of the Inner Wall

**Figure 9:**
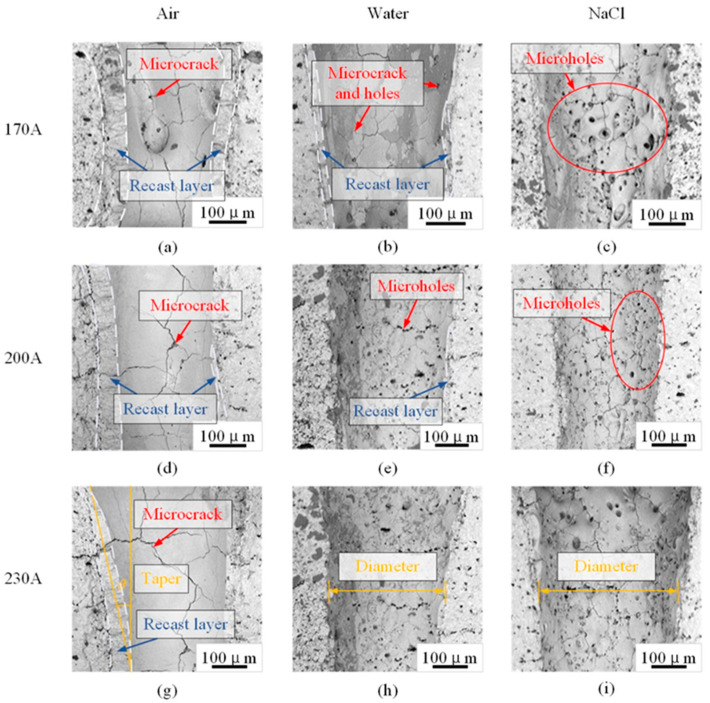
Morphology of holes’ inner walls (drilling in air, water, and NaCl solution). Subfigure (**a**–**i**) show the morphologies of different drilling conditions (The same current and same environment are displayed in a row and a column respectively).

Figure 9 shows the inner wall morphology of laser drilling in three environments (air, water, and NaCl solution) under current conditions of 170, 200, and 230 A.

Figure 9 shows the morphology of the holes’ inner walls. Compared to drilling in a solution, the inner wall of the hole drilled in air was covered with a thick, accumulated recast layer, which was reflected as a flat, cracked, non-porous surface. However, the recast layer was an unstable surface which was easy to peel off under friction and impact, which directly affected the working environment of the workpiece. Therefore, the generation of a recast layer should be minimised as much as possible. The thickness of the recast layer on the hole wall drilled in the solution was significantly reduced. When drilled in the solution environments, flowing liquid cooled down the melted slag and removed it from the surface of the inner wall.

Comparing the morphology of the inner wall of the holes drilled in the water and NaCl solution, it can be seen that the thickness of the recast layer in the NaCl solution was smaller. The mechanical effect of the laser in liquid was that the laser focused on the surface of the material, which heated up and formed a jet of material. The sprayed material generated a back-impact force on the surface of the material, while expanding outwards and compressing the surrounding solution to form a shock wave. However, the impact force of the millisecond-level laser on materials in a neutral salt solution was much greater than that in water [26]. Therefore, when drilling in the NaCl solution, slag could be better removed and its adhesion and accumulation on the hole wall was reduced. Under the action of high-temperature laser energy, the liquid produced a cavitation effect, which burst and hit off the recast layer from the inner wall. At the same time, the cavitation effect formed a number of micro-pores in the inner wall.

**Figure 10:**
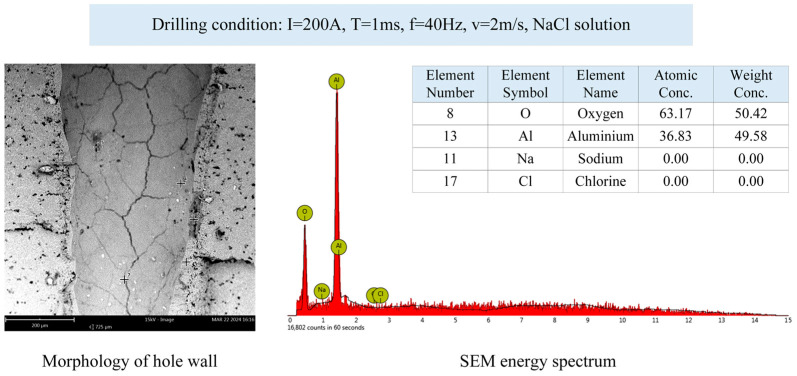
The SEM energy spectrum of the hole wall.

In order to study the effect of the NaCl solution on the chemical composition of hole walls, the SEM energy spectrum is shown in Figure 10. Drilling in water and NaCl solutions did not affect the chemical composition of the inner wall, because the material alumina ceramic did not react with a neutral salt NaCl solution or water. And during the laser drilling process, the solution was not electrified.

The current values also affected the quality of the hole. If the current was as low as 170 A, the cavitation effect of the liquid was not strong enough to discharge the recast layer. As the current and power of the laser pulse increased, the evaporation pressure inside the hole increased, which helped the liquid-phase material inside the hole to be effectively discharged. In addition, the higher current accelerated the convection of the liquid, causing the liquid to flush away the recast layer at a faster rate and carry away the slag. Therefore, the thickness of the recast layer on the hole wall drilled in the solution was relatively small at a current of 200 A and 230 A.

#### 3.2.4. Drilling Mechanism and Process

**Figure 11:**
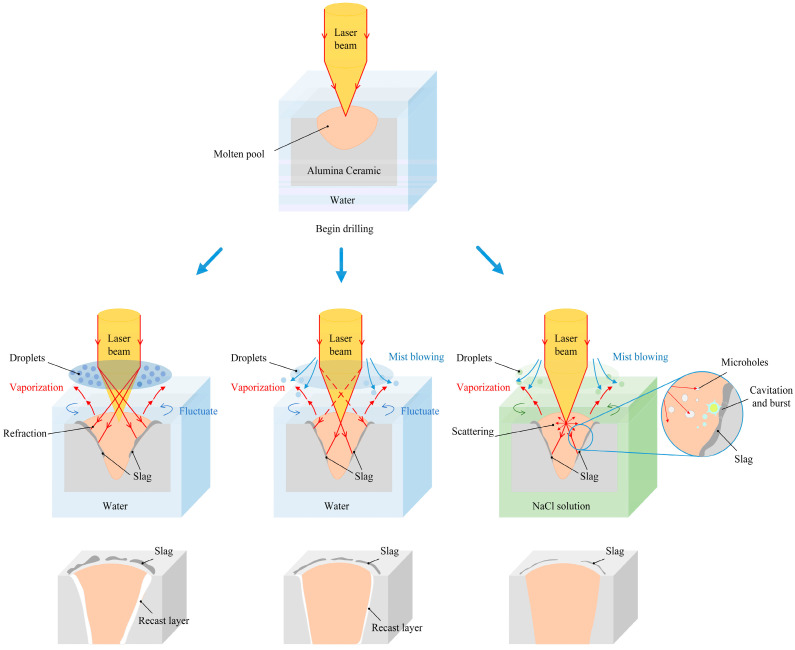
Drilling mechanism in water and NaCl solution.

As shown in Figure 11a, when laser drilling in water, the laser beam needs to pass through a solution surface and water vapour layer generated by the heat absorption and evaporation of water. It causes refraction and weakening of the laser energy, which will lead the hole quality to have a large taper and low roundness. The water can take away most of the slag, and a small part of the molten layer is attached to the hole wall.

Considering the method of mist blowing shown in Figure 11b, the mist droplets formed by the water evaporation were blown away, and the weakening degree of the laser during transmission was reduced. More energy was used for drilling, which significantly improved the taper and roundness of the holes. The wind and the fluctuation of water jointly promoted heat dissipation, which further thinned the molten layer and obtained better hole quality.

Figure 11c shows a changed composition of the solution. Due to the mechanical effect of the laser in the NaCl solution, the impact force on the material was much greater than that in water. At the same time, the solution cavitation effect, generated by the absorption of laser energy, caused an explosive impact on the molten material adhered to the surface of the hole wall. The hole wall had almost no recast layer and ideal roundness and taper.

Based on the above analysis, under reasonable parameters and processing environments, the quality of laser drilling can be improved. The experimental results of our work indicate that under the conditions of f=40 Hz and T=1 ms, the optimal processing parameters are I=200 A, v=2 m/s, and drilling in a NaCl solution environment.

## 4. Conclusions

During the process of the conventional water solution-assisted laser drilling of alumina ceramic, the vapour droplets refract and scatter the laser beam, which weakens the laser energy and reduces the quality of drilling. This work aimed to disperse evaporated suspension droplets and reduce the influence of laser refraction and scattering. The main conclusions are as follows:

(1) By experimenting with different solutions in the assisted laser-drilling of alumina ceramic under different wind speed conditions, it was found that the wind-blowing method with an appropriate speed can effectively improve the taper and roundness of the hole. This was because the weakening degree of the laser during transmission was reduced by wind, and the fluctuation of water jointly promoted the heat dissipation. The optimal wind speed was found to be 2 m/s. While excessive wind speed will lead to a decrease in hole taper and roundness, because the laser is scattered and refracted again, the solution fluctuated and scattered the laser beam in stronger wind speed. At a low wind speed of 0.0–1.5 m/s, the droplets were not dispersed enough, and the laser beam was extensively scattered and refracted.

(2) When the current was 200 A, the optimal hole taper and roundness could be obtained, whether drilling in a water or NaCl solution. In the excessive current range of 220–260 A, the laser energy was too high, and a large amount of water was vapoured in the solution. In the small current range of 160–190 A, the current was too small to change the taper of the hole, whether in the water or NaCl solution.

(3) In different environments, the thickest recast layer was found on the wall of the hole created by laser drilling in air. The recast layer was obviously thinner in the other two solutions because the solutions produced a cavitation effect, which burst and hit off the recast layer from the inner wall. The quality of the hole drilled in the NaCl solution was the best. The laser drilling process can easily achieve a more ideal hole taper with processing parameters of a current of 200 A and a wind speed of 2 m/s.

## Figures and Tables

**Table 1:** Laser parameters.

Parameters	Values
Maximum power of current output	12 KW
Average output power	300 W
Maximum single-pulse output energy of laser beam	120 J
Wavelength	1064 nm
Pulse width (T)	1 ms
Frequency (f)	40 Hz
Focusing position	−0.5 mm
Spot diameter	3 mm

**Table 2:** Experimental parameters.

Parameters	Values
Drilling environment	Air, water, NaCl
Wind speed (*v*)	0, 0.5, 1, 1.5, 2, 2.5, 3, 3.5, 4, 4.5 (m/s)
Laser current (I)	170 A, 200 A, 230 A
Drilling depth (h)	1 mm

## Data Availability

The raw data supporting the conclusions of this article will be made available by the authors on request.
